# Supplementary material for: The interactome of intact mitochondria by cross-linking mass spectrometry provides evidence for coexisting respiratory supercomplexes
Source: Mol Cell Proteomics. 2017 Dec 8;17(2):216–32. doi: 10.1074/mcp.RA117.000470 (PMC5795388; doi:10.1074/mcp.RA117.000470)
Supplement: Supplemental Data [file supp_RA117.000470_133922_0_supp_23978_fzffwf.zip › spectra_annotation/mito_spectra_annotation/444-1-8-1-11-1.pdf]

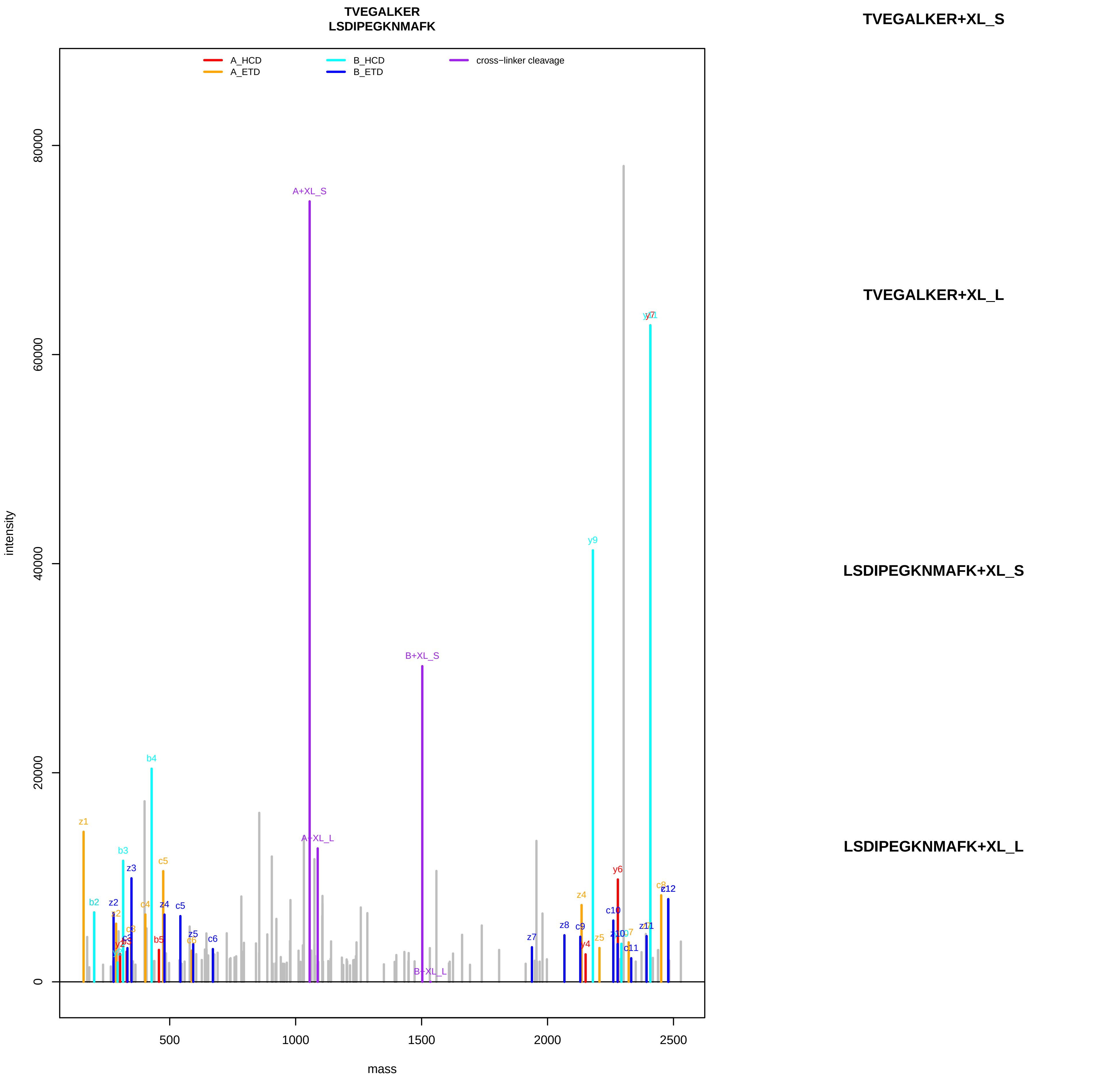
TVEGALKER
LSDIPEGKNMAFK
TVEGALKER+XL_S
TVEGALKER+XL_L
LSDIPEGKNMAFK+XL_S
LSDIPEGKNMAFK+XL_L
A_HCD
A_ETD
B_HCD
B_ETD
cross−linker cleavage
A+XL_S
B+XL_S
A+XL_L
B+XL_L
intensity
mass
0
20000
40000
60000
80000
500
1000
1500
2000
2500
z1
b2
z2
b3
z3
b4
c4
z4
c5
b5
c6
z5
z7
z8
c9
z4
z5
y4
y9
y6
c10
z10
c11
z11
c8
c12
y7
